# Supplementary material for: Targeting the Human Influenza a Virus: The Methods, Limitations, and Pitfalls of Virtual Screening for Drug-like Candidates Including Scaffold Hopping and Compound Profiling
Source: Viruses. 2023 Apr 26;15(5):1056. doi: 10.3390/v15051056 (PMC10221779; doi:10.3390/v15051056)
Supplement: Supplementary file 1 [file viruses-15-01056-s001.zip › viruses-2237917-supplementary.pdf]

viruses

Supplemental Material (SM)

1) Binding pattern analyses for **pharmacophore modeling** for the reference compounds with text and figures.

2) Modeling details and settings for the **virtual screening** with text and figures.

1) Binding pattern analyses for **<u>pharmacophore modeling</u>** for the reference compounds with text and figures.

We analyzed each binding mode individually. At this stage of work, we focused on the four N1 inhibitors and the natural substrate occupying the binding site of viral protein N2 (**Figure S1).** Panels A, B, C and D of Figure S1 display oseltamivir, zanamivir, peramivir and laninamivir in interaction with their specific residues at the binding site of target N1 [25]. Sialic acid interacts with N2 residues as depicted in Panel E. For instance, Pharmacophore modeling **was** guided by the graphical analysis of Panel A through E. For instance, the interacting amino acid residues at N1 active site and distinct atoms of reference drug oseltamivir (**Table S1**). As outlined in the main text, the acetamido function was reported to be of outmost importance for receptor recognition [39] (cf. Panels B, C, D, or E, too). The inlay of Panel A highlights the acetamido – Arg152 interaction as it was experimentally observed in the published oseltamivir-liganded crystal complex (PDB entry 3CL0). As a proof of concept for AD4 is our blind docking of AAmol with its acetamido function which interacts with arginine Arg152, too (**Figure 6**).

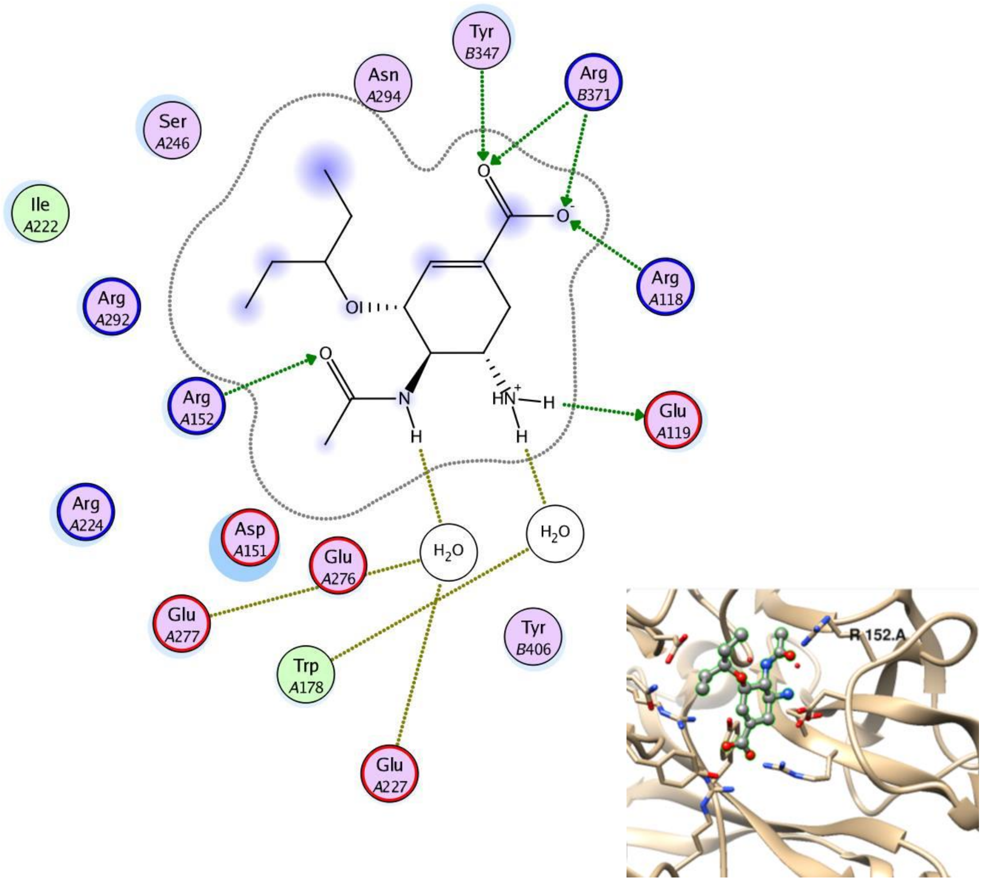

Figure S1. Panel A. Reference ligand oseltamivir interaction**.**

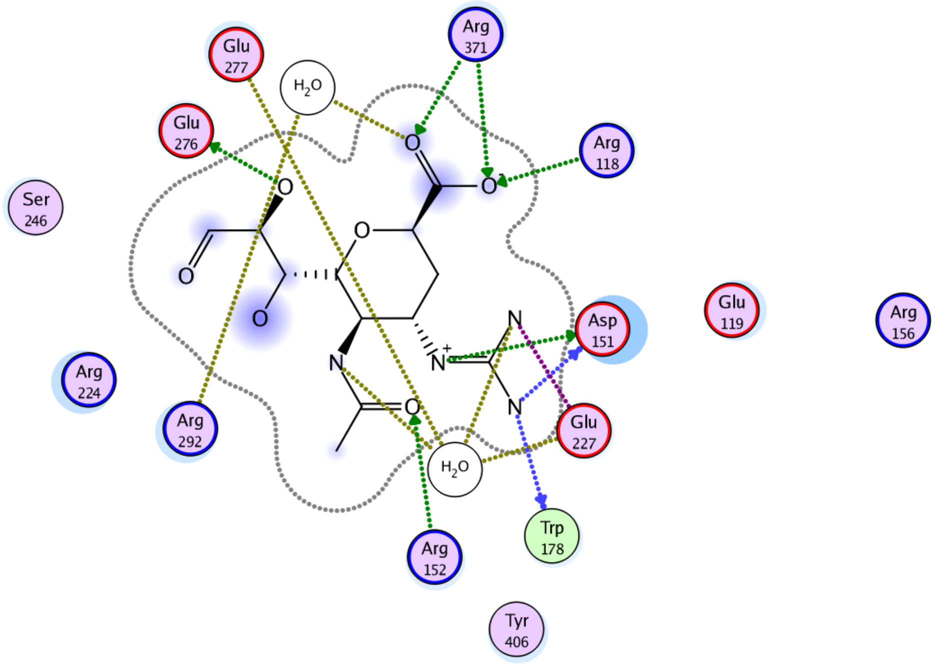

Figure S1. Panel B. Reference ligand zanamivir interaction. 2D projection of the interaction between amino acids and zanamivir occupying the N1 active site.

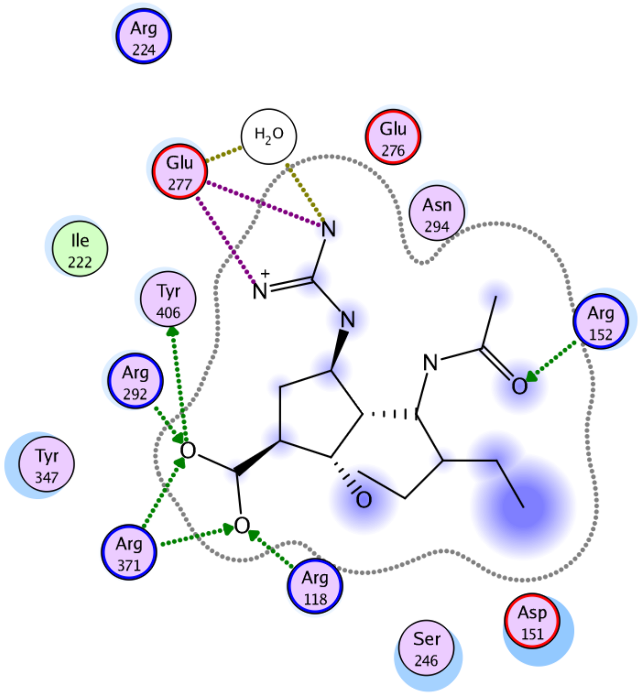

Figure S1. Panel C. Reference ligand peramivir interaction. 2D projection of the interaction between amino acids and peramivir occupying the N1 active site.

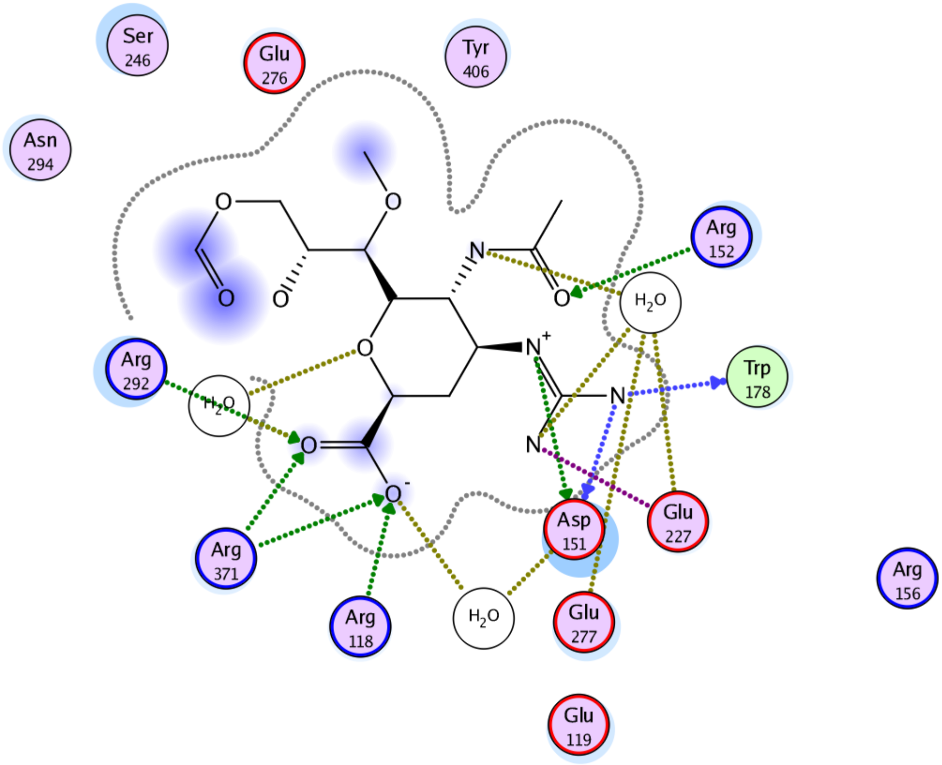

Figure S1. Panel D. Reference ligand laninamivir interaction. 2D projection of the interaction between amino acids and laninamivir occupying the N1 active site.

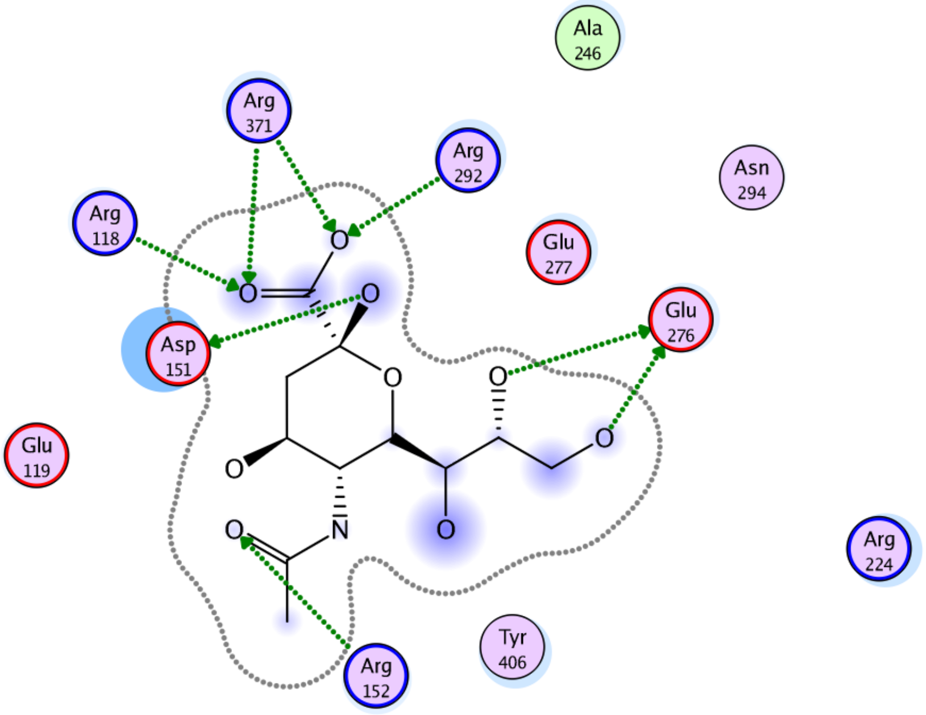

Figure S1. Panel E. Reference natural substrate sialic acid interaction. 2D projection of the interaction between amino acids and sialic acid occupying the N2 active site. Of note, the binding mode between acetamido function and residue Arg152 remains conserved when crossing from viral protein N1 to N2.

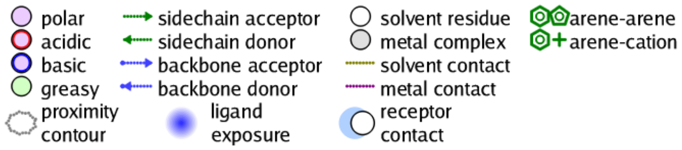

Figure S1. Panel F. Graphical symbols for ligand interaction. In general ligand-receptor molecular interactions involve intermolecular association forces that can be hydrophobic interactions, Van Der Waals interactions, hydrogen bonds, and electrostatics which holds also for the interacting amino acids at the binding site.

Table S1: Oseltamivir interaction. Listing of interacting amino acids at the N1 active site and inhibitor oseltamivir.

**Amino acids of N1 (3CL0 numbering)**	**Atom or group of ligand (OS)**	**Approximated distance (Å)**	**Type of interactions**
Arg118, Arg292, Arg371	carboxylate group	1.7-3	salt bridge & hydrogen bond
Tyr347, Tyr406	carboxylate group	3-3.6	hydrogen bond
Glu119, Asp151, Glu227	amino group	2-3	salt bridge & hydrogen bond
Arg152	amide group	3	hydrogen bond
Glu227, Glu276, Glu277	NH of amide group	Interaction by H_20 (2.5)	hydrogen bond
Trp178	amide group	4	hydrophobic (by C-H/π) forces
Ile222	amide group	2-6	Hydrophobic
Glu276, Arg224	pentyl ether	2-6	London dispersion forces
Tyr274, Ile222, Ala246	pentyl ether	2-6	hydrophobic forces

STRUCTURE – ACTIVITY RELATIONSHIPS

Oseltamivir relevant substructure embraces an aliphatic aromatic cycle, the carboxyl group in the C1 position and the acetamide in the C4 position, however the hydroxyl groups that were present in sialic acid are exchanged for a 1-ethylpropoxy ether at C3.

Zanamivir´s overall structure resembles that of the natural substrate, only changing the C4 hydroxyl group to a guanidino group, therefore it is expected to have a similar affinity towards the viral enzyme and possibly the guanidino group nitrogen atoms form ionic interactions with acidic amino acids. They can also establish hydrogen bonds through primary amines.

Peramivir also keeps an aliphatic aromatic ring, the carboxyl group and a 2-ethylbutyl acetamide, it also contains a guanidino group with primary amines on it for hydrogen bonding with amino acid residues or (crystal) water.

Laninamivir contains the same structure as zanamivir, however it presents a methoxy group at C7, another hydroxyl group at C8 and an ester bridge at C9. They can form hydrogen bonds like sialic acid with its hydroxyl group.

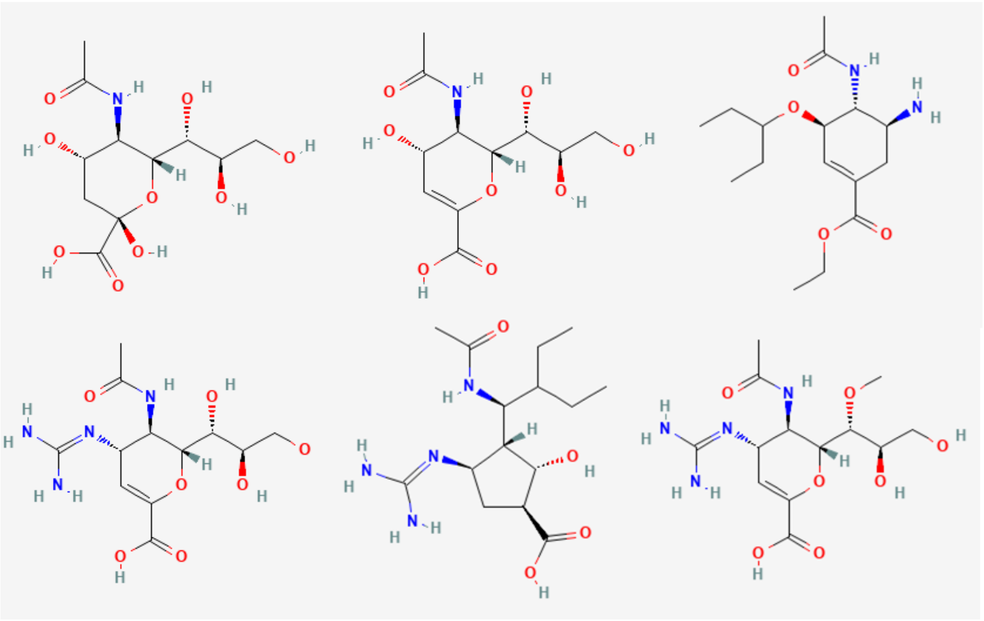

Figure S2. Stereochemical drawing of natural substrate with the five reference ligands. The order is the same as in Figure 2: in the top row, from left to right: sialic acid, DANA and oseltamivir. In the bottom row, from left to right: zanamivir, peramivir and laninamivir. Color code: black C atoms, white H atoms, red O atoms, blue N atoms, respectively.

<u>Stereochemical and structural data sources:</u>

Sialic Acid:
National Center for Biotechnology Information (2023). PubChem Compound Summary for CID 445063, 5-N-Acetyl-beta-D-neuraminic acid. Retrieved March 9, 2023 from https://pubchem.ncbi.nlm.nih.gov/compound/5-N-Acetyl-beta-D-neuraminic-acid.

DANA:
National Center for Biotechnology Information (2023). PubChem Compound Summary for CID 65309, 2-Deoxy-2,3-dehydro-n-acetyl-neuraminic acid. Retrieved March 9, 2023 from https://pubchem.ncbi.nlm.nih.gov/compound/2-Deoxy-2_3-dehydro-n-acetyl-neuraminic-acid.

National Center for Biotechnology Information (2023). PubChem Compound Summary for CID 65028, Oseltamivir. Retrieved March 9, 2023 from https://pubchem.ncbi.nlm.nih.gov/compound/Oseltamivir.

National Center for Biotechnology Information (2023). PubChem Compound Summary for CID 60855, Zanamivir. Retrieved March 9, 2023 from https://pubchem.ncbi.nlm.nih.gov/compound/Zanamivir.

National Center for Biotechnology Information (2023). PubChem Compound Summary for CID 154234, Peramivir. Retrieved March 9, 2023 from https://pubchem.ncbi.nlm.nih.gov/compound/Peramivir.

National Center for Biotechnology Information (2023). PubChem Compound Summary for CID 502272, Laninamivir. Retrieved March 9, 2023 from https://pubchem.ncbi.nlm.nih.gov/compound/Laninamivir.

2) Modeling details and settings for the **virtual screening** with text and figures.

In the retrospective virtual screening, known active compounds are used, in order to validate the screening tools on the specific chemistry of the biomolecular target of study and to select the best parameters with which these tools should be used (calibration).

Construction of the test library for retrospective virtual screening

For the construction of the test virtual library, 10 positive and 23 negative controls with similar properties were selected. The positive controls are made up of the molecules used for the construction of the 2D (fingerprint) and 3D (pharmacophore) filters, and correspond to the ligands that were extracted from the following PDB IDs: sialic acid (2BAT), oseltamivir (3CL0, 2HU0), peramivir (2HTU), zanamivir (2B7E), and DANA (2HTR). More details are provided in Table 1. As presumably inactive negative controls, 23 molecules were selected that were obtained from PubChem [14] by performing a search for structures with properties similar to those calculated from the active positive controls (**Table S2).**

Table S2: Library test characteristics. Properties of active (positive controls) and inactive (negative controls) molecules selected for construction of the virtual library testing (retrospective VS).

**Properties**	**Controls: positive = active**		**Controls: negative = inactive**	
	**Mean value**	**standard deviation**	**Mean value**	**standard deviation**
**Molecular Weight**	297	46	312	11
**H-Bond Acceptors**	3	2	5	2
**H-Bond Donors**	3	2	3	1
**Acidic atoms**	2	0	1	2
**Basic atoms**	2	1	1	1
**Hydrophobic atoms**	10	3	9	3
**Rotatable bonds**	7	2	7	2

All screening control and decoy moieties which have been introduced into the screening test are listed in **Table S3**.

Table S3: Control structures. Positive and negative control models selected for the construction of the virtual test library.

Positive controls	Negative controls (col. 1)	Negative controls (col. 2)
Oseltamivir (3CLO and 2HU0) 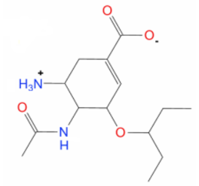	CID10040785 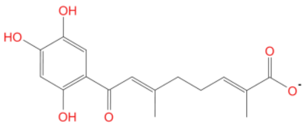	CID40637266 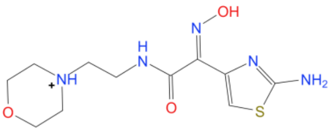
Zanamivir (2B7E) 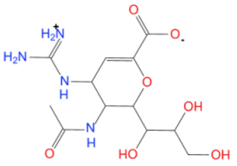	CID18659129 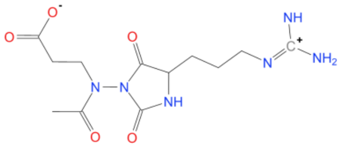	CID46268586 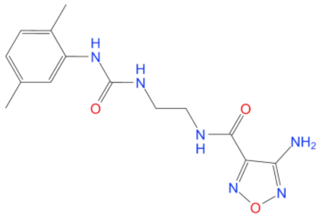
Sialic acid (2BAT) 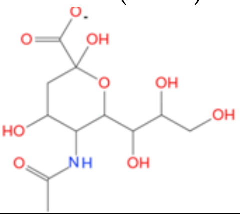	CID18719644 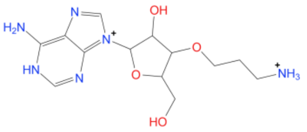	CID46318264 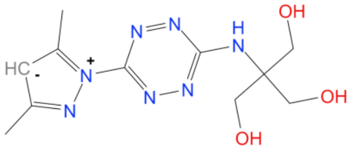
Peramivir (2HTU) 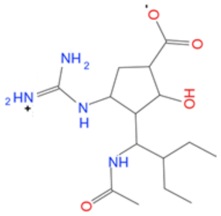	CID18740642 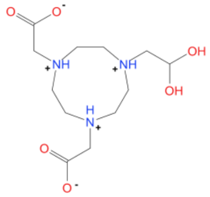	CID4892543 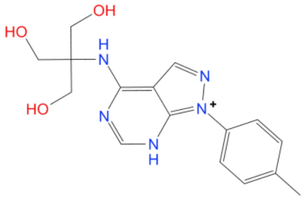
DANA (2HTR) 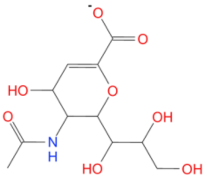	CID18742848 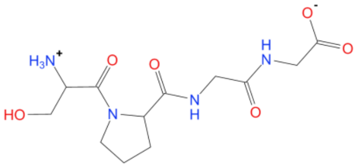	CID558227 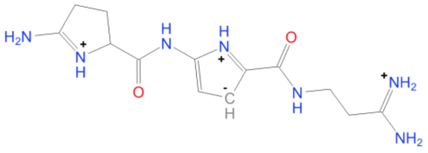
BANA113 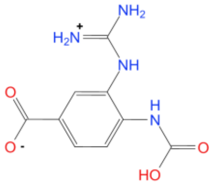	CID21191623 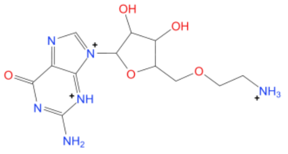	CID6563843 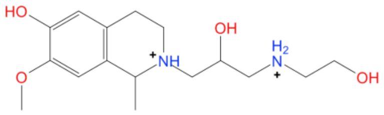

G20 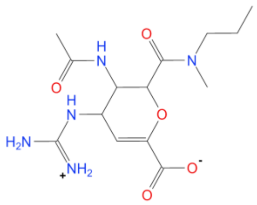	CID23659933 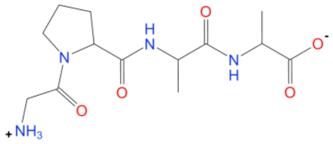	CID687723 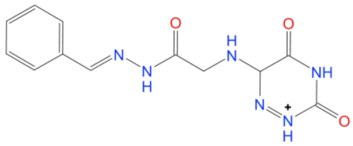
BANA106 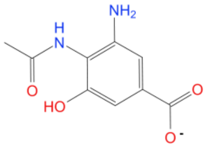	CID290835 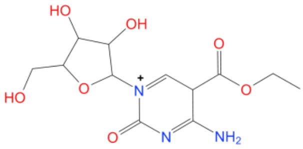	CID8142923 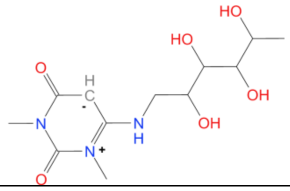
BCX1898 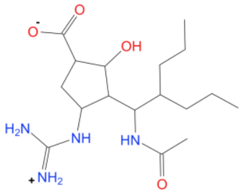	CID299983 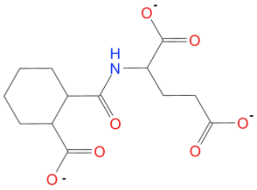	CID836333 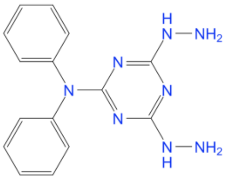
	CID300677 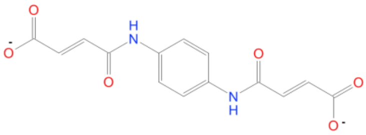	CID9658365 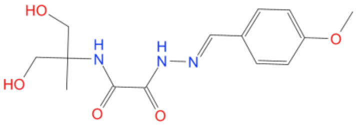
	CID3082122 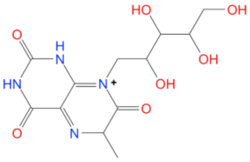	CID40605908 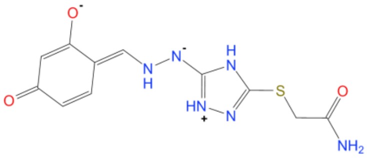
	CID3086657 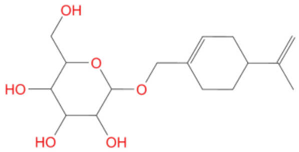	

The virtual drug library for prospective virtual screening

660,961 molecular entries were screened in the Molecular Operating Environment database [18]. It constitutes a collection from different sources: Chemdiv, Biofocus, ASDI, Bionet, BiotechCA, Chembridge, Chemical block, Asinex, Cerep, ChemStar, As Synthese-Biotech, Aurora, Akos, , Art-Chem, EMC Microcollectios, Lithuania, FCHC, Comgenex, Life Chemicals, InterBioScreen, Labotest, InnovaPharma, Enamine, Otava, Peakdale, Toslab, MDPI, Chem T&I, Tripos, Specs, Chem T&I, Maybridge, BTB, Menai, Sigma Aldrich, Scientific Exchange, Nanosyn , Princeton biomolecular, Pharmeks, Scientific exchange, Pyxis Discovery, Sigma Aldrich SALOR, TimTec, Vitas-M-Lab [18].

Additional details on construction and calibration of the 2D filter (fingerprint)

Several 2D fingerprint models were constructed from the two-dimensional features of the following ligands: Sialic acid (2BAT), Oseltamivir (3CL0), Peramivir (2HTU), Zanamivir (2B7E) and DANA (2HTR). The chosen metrics was the TGD type (Typed Graph Distance), since it encodes the fundamental molecular information for the interaction of a ligand with its receptor (donor, acceptor, polar, anion, cation, hydrophobic [18]. Twenty-four fingerprint models were designed using the Tanimoto coefficient as a similarity metric (**Table S4**). Changing the overlap values and minimum matching requirements of the scoring function helped analyze the virtual data set with the fingerprint models. The minimum matching requirements refer to the required minimum number of model fingerprints that must match the fingerprints of each of the molecules contained in the database to result in a hit. There is coincidence in an individual comparison of similarity between the model and the evaluated molecule when said comparison exceeds the threshold value of overlap previously defined. Overlap refers to the percentage of similarity used to determine if the fingerprint of the tested molecule matches a specific fingerprint in the model [18]. In the next step, the TGD type models were calibrated with the enriched data set.

Assessment metrics used in retrospective virtual screening.

The characteristics of the filters designed and the results obtained after carrying out the retrospective virtual screening of each fingerprint model in the virtual library are presented in **Table S4**. Here the objective was to determine which is the most appropriate model, that is, the one that is capable of adequately discriminating between active and inactive molecules. Here a test set was created with 10 active and 23 inactive decoys, so the maximum enrichment factor (EF) that could be obtained in the generated models was the ratio (10+23) / 10 = 3.3.

Table S4: Enrichment factors. Listing of the enrichment metrics for the fingerprint models. After designing them they were calibrated in the virtual test library and tested to see which best fits the data. #: model number, CM (%): minimum match percentage, T(%): overlap percentage, D: total number of molecules in the library, A: number of active molecules in the library, Ht: number of molecules in the hit list obtained by screening a library with the model under study, Ha: number of active molecules in the hit list obtained by screening a library with the model under study, FN: false negatives, FP: false positives, %Y : asset return, %A: asset ratio, EF: enrichment factor, GH: hit list goodness.

# **Modelo**	**CM (%)**	**T (%)**	**D**	**A**	**Ht**	**Ha**	**FN**	**FP**	**%Y**	**%A**	**EF**	**GH**
**1**	80	80	33	10	0	0	10	0	0.00	0	0	0
**2**	75	80	33	10	0	0	10	0	0.00	0	0	0
**3**	65	80	33	10	0	0	10	0	0.00	0	0	0
**4**	80	70	33	10	2	2	8	0	100	20	3.30	0.80
**5**	70	70	33	10	2	2	8	0	100	20	3.30	0.80
**6**	10	80	33	10	7	7	3	0	100	70	3.30	0.93
**7**	10	10	33	10	33	10	0	23	30.30	100	1.00	0
**8**	20	80	33	10	7	7	3	0	100	70	3.30	0.93
**9**	30	85	33	10	2	2	8	0	100	20	3.30	0.80
**10**	30	80	33	10	3	3	7	0	100	30	3.30	0.83
**11**	30	70	33	10	9	9	1	0	100	90	3.30	0.93
**12**	0	85	33	10	33	10	0	23	30.30	100	1.00	0
**13**	25	80	33	10	3	3	7	0	100	30	3.30	0.83
**14**	25	70	33	10	9	9	1	0	100	90	3.30	0.98
**15**	20	70	33	10	9	9	1	0	100	90	3.30	0.98
**16**	20	65	33	10	15	10	0	5	66.67	100	2.20	0.60
**17**	20	68	33	10	11	10	0	1	90.91	100	3.00	0.90
**18**	25	65	33	10	10	10	0	0	100	100	3.30	1.00
**19**	25	68	33	10	9	9	1	0	100	90	3.30	0.98
**20**	30	65	33	10	10	10	0	0	100	100	3.30	1.00
**21**	35	65	33	10	10	10	0	0	100	100	3.30	1.00
**22**	40	65	33	10	10	10	0	0	100	100	3.30	1.00
**23**	45	65	33	10	9	9	1	0	100	90	3.30	0.98
**24**	50	65	33	10	9	9	1	0	100	90	3.30	0.98

Based on the results obtained from the retrospective virtual screening, the fingerprint model number 24 (40% minimum coincidence and 65% overlap) was chosen to carry out the prospective virtual screening since it is the one that presented the best scoring filter metrics (Y results, %A, EF and GH). Number 24 model allowed us to adequately discriminate the 10 active molecules from the 23 inactive ones. Of note, exhaustive data analysis revealed that models 18, 20 and 21 also achieved the same results as 22, however the latter was chosen because it is stricter in terms of the minimum percentage of coincidence necessary to filter the database, in such a way that the filter is more selective.

2D prospective virtual screening

Prospective virtual screening was performed using the fingerprint model number 24.

Additional details on construction and calibration of the 3D filter (pharmacophore)

To determine the molecules that will be included in the construction of the pharmacophoric models, a superposition of the active conformations of the selected ligands was performed: Sialic acid (2BAT), Oseltamivir (3CL0, 2HU0, 3CL2), Peramivir (2HTU), Zanamivir (2B7E, 3CKZ) and DANA (2HTR, 2HTW) and the RMSDs between the different conformations of the same ligand were calculated (**Table S5**). Various publications define that an RMSD value between 0 and 2.5 indicates that there are no significant differences between the overlapping molecules, as this RMSD range represents the experimental precision of protein X-ray crystallography [S46].

Table S5: Positional evaluation (RMSD). Self or back docking trials and spatial comparison of the final poses to the internal reference ligand (Basis). RMSD: root mean square distance, is a measure of proximity between two molecules, being one the internal reference (Basis). RMSD values < 0.5 to 1 reflect excellent results, and values < 2 are acceptable for small organic compounds.

**PDB ID**	**Ligand**	**Type NA**	**RMSD**
3CL0 (*)	Oseltamivir	N1, H274Y	Basis
3CL2	Oseltamivir	N1, N294S	0.5
2HU0	Oseltamivir	N1, H253Y	0.6
3B7E	Zanamivir	N1	0
3CKZ	Zanamivir	N1, H274Y	0.1
2HTU	Peramivir	N8	0
2HTR	DANA	N8	0
2HTW	DANA	N4	0.4
2BAT	Sialic acid	N2	0

Models were built from 3D alignment of selected molecules (**Figure S3**). The characteristics selected for the design of the models were the following: anion, cation, hydrogen bond donor (HBD, H-bond donor), hydrogen bond acceptor (HBA, H-bond acceptor), hydrophobic. These characteristics were chosen because they are the ones that are present and allow the interaction of the ligands with the enzyme. This was deduced based on the analysis of the target with the reference ligands. Additionally, an analysis furthered the understanding of all those features that were consistent with the specified set of conformations of the aligned ligands and their locations were defined from the crystallographic coordinates of the selected atoms or groups (**Figure S3**). From this analysis, the models were designed, the radii were assigned manually in such a way that the characteristics of the molecules used for the construction of the models were included.

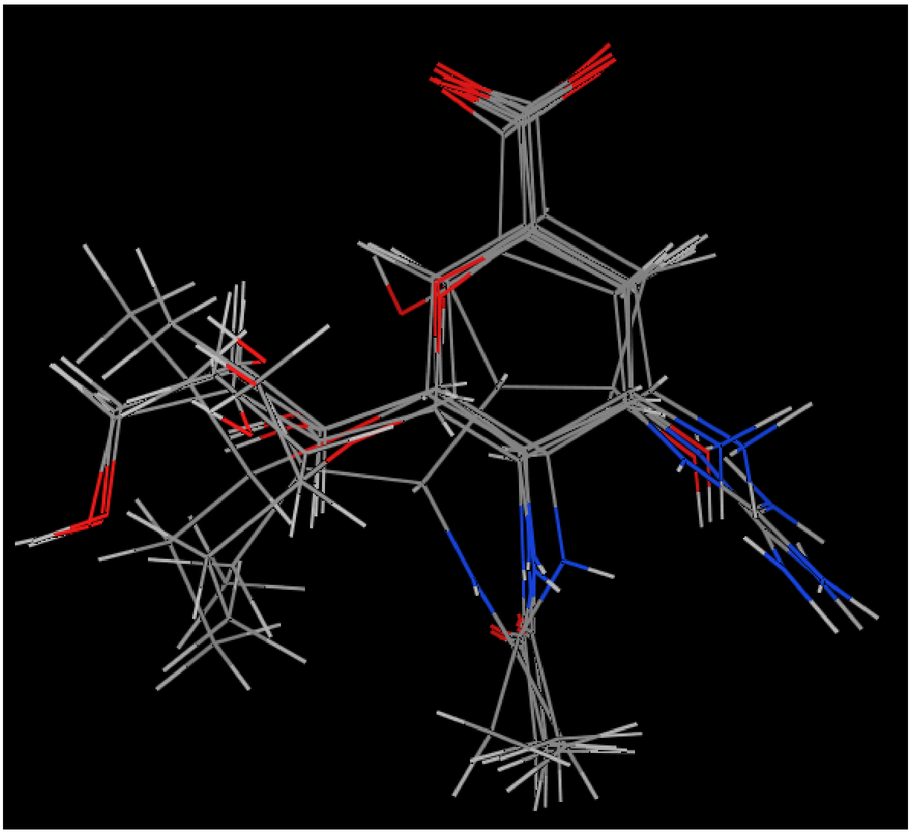

Figure S3. Ligand superposition for pharmacophore construction. 3D alignment of ligands with the active conformations which were relevant for the construction of the pharmacophore models.

Based on the calibration results pharmacophore model 24 was chosen to perform the prospective 3D virtual screening (**Figure S4**). It was the one that allowed us to discriminate all ten active molecules from the 23 inactive control compounds. Moreover model 24 obtained the best metrics score during evaluation.

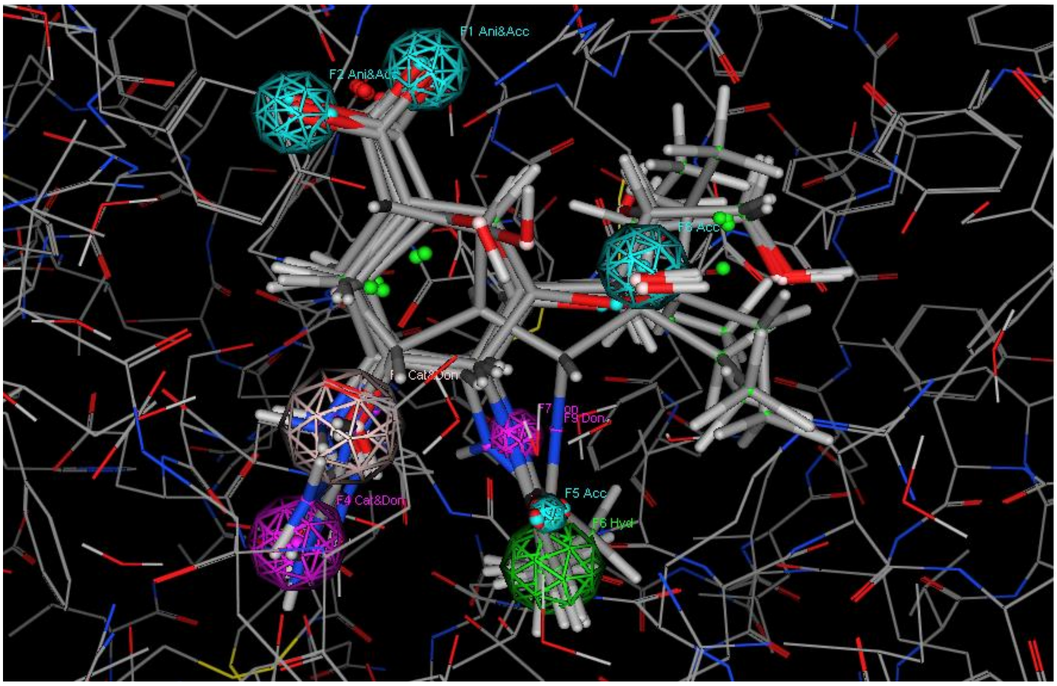

Figure S4. Pharmacophore model. Graphic representation of the pharmacophoric model 24 selected to carry out prospective virtual screening. The spheres mark the exact location of the 3D fingerprints which taken together represent the pharmacophore. Color code: light blue or magenta spheres: oxygen or nitrogen for hydrogen bonding; green spheres for hydrophobic attraction; grey sphere symbolizes a required volume. (Details in SM, labeling omitted.)

At this stage of work, pharmacophoric modeling underwent calibration tests using the virtual test library for retrospective virtual screening. To perform retrospective and prospective virtual screening with a 3D filter, it is necessary to have a conformational database, so some possible conformations of the test molecules were calculated. To determine the optimal parameters for the calculation of conformations, several tests were carried out changing the residual strain energy limit in local minimum (strain energy), maximum number of conformations and RMSD to remove duplicates. In all cases, the versatile MMFF94X force field was used because it had been calibrated for small organic molecules and proteins alike. Finally, the calculation protocol was applied to obtain an appropriate conformational space. For details, see section Preparation of virtual libraries for 3D filtering – conformational calculation. For this purpose, a total of 17961 conformations of the 33 test set molecules were generated. Their active conformations were used for model construction, simply adding them for filter calibration testing. Twenty-four pharmacophoric models were built during evaluation studies in the virtual test library (for details see the following tables below, especially **Table S6** which was split into several parts to accommodate the 24 models into table columns).

Table S6: Pharmacophore models. Characteristics of the designed pharmacophoric models, the pharmacophoric characteristics of each model are indicated, as well as their location and radius expressed in Ǻ. The E indicates that the indicated feature was considered essential for that particular model. Partial match (CP) requirements of the models are presented. Abbreviations; Ani&Acc: anion and hydrogen bond acceptor; Cat&Don: cation and hydrogen bond; Acc: hydrogen bond acceptor; Hid: hydrophobic; Gift: hydrogen bond donor.

		**# Model of pharmacophore**								
			1	2	3	4	5	6	7	8
**Caracterı́stics**	**Center**	**R(Å)**								
**Ani&Acc**	**O carboxyl**	**0.8**	✓	✓	✓	✓	✓	✓E	✓	✓
**Ani&Acc**	**O carboxyl**	**0.8**	✓	✓	✓	✓	✓	✓	✓	✓
**Cat&Don**	**N+ group amino**	**1**	✓	✓	✓	✓	✓E	✓	✓	✓
**Cat&Don**	**N+ guanidine**	**1.2**	✓	✓	✓	✓	✓	✓	✓E	✓
**Acc**	**O amide**	**0.78**	✓	✓	✓	✓	✓	✓	✓	✓E
**Hid**	**CH3 amide**	**0.9**	✓	✓	✓	✓	✓	✓	✓	✓
**Don**	**N amide**	**1.19**	✓	✓	✓	✓	✓	✓	✓	✓
**Acc**	**O pentyl ether, OH glycerol**	**1.06**	✓	✓	✓	✓	✓	✓	✓	✓
**Coincidence is partial (CP)**			×	7	6	5	5	5	5	5

Table (continue) Evaluation of pharmacophore models (number 9 to 17).

			# Model of pharmacophore								
			9	10	11	12	13	14	15	16	17
**Característics**	**Center**	**R(Å)**									
**Ani&Acc**	**O carboxyl**	**0.8**	✓	✓	✓	✓E	✓E	✓E	✓E	✓E	✓E
**Ani&Acc**	**O carboxyl**	**0.8**	✓	✓	✓	✓E	✓E	✓E	✓E	✓E	✓E
**Cat&Don**	**N+ Group amino**	**1**	✓	✓	✓	✓	✓	✓	✓	✓	✓
**Cat&Don**	**N+ guanidine**	**1.2**	✓	✓	✓	✓	✓	✓	✓	✓	✓
**Acc**	**O amide**	**0.78**	✓	✓	✓	✓	✓	✓	✓E	✓	✓
**Hid**	**CH3 Amide**	**0.9**	✓E	✓	✓	✓	✓E	✓	✓	✓	✓
**Don**	**N amide**	**1.19**	✓	✓E	✓	✓	✓	✓E	✓E	✓E, Don&Acc	✓E
**Acc**	**O pentyl ether, OH glycerol**	**1.06**	✓	✓	✓E	✓	✓	✓	✓	✓	✓E, Don o Acc
**Coincidence is Partial (CP)**			5	5	5	5	5	5	5	5	5

Table (continue) Evaluation of pharmacophore models (number 18 to 24).

# Model of pharmacophore															
		R	18	R	19	R	20	R	21	R	22	R	23	R	24
	Center														
Ani &Acc	O carb.	0.7	✓E	0.7	✓E	0.7	✓E	0.7	✓E	0.7	✓E	0.7	✓E	0.7	✓E
Ani &Acc	O carb.	0.7	✓E	0.7	✓E	0.7	✓E	0.7	✓E	0.7	✓E	0.7	✓E	0.7	✓E
Cat &Don	N+ Group amino	1	✓	1	✓	1	✓	1	✓	1	✓	1	✓	1	✓
Cat &Don	N+ Guanidi-ne	1.2	✓	1.2	✓	1	✓	1	✓	1	✓	1, M	✓	1, M	✓
Acc	O amide	0.78	✓	0.78	✓	0.5	✓	0.3	✓	0.3	✓	0.3	✓	0.3	✓
Hid	CH3 Amide	0.9	✓	0.9	✓	0.85	✓	0.85	✓	0.85	✓	0.85	✓	0.85	✓
Don	N amide	0.85	✓E	0.47	✓	0.47	✓	0.47	✓	0.47	✓	0.47	✓	0.47	✓

Acc	O pentyl ether, OH glycerol	1.06	✓	1.06	✓	1.06	✓	1.06	✓	1.06	✓	1.06	✓	0.73, M	✓
Don	N amide Peramivir			0.3	✓	0.3	✓	0.3	✓	0.1	✓	0.1	✓	0.1	✓
CP			5		5		5		5		5		5		5

Pharmacophoric models were designed and calibrated in the enriched test library. These results were also documented in a listing (**Table S7**).

Table S7: Performance evaluation. Listing of the evaluation process for pharmacophore models. Abbreviations: #: model number, CM (%): minimum match percentage, T(%): overlap percentage, D: total number of molecules in the library, A: number of active molecules in the library, Ht: number of molecules in the hit list obtained by screening a library with the model under study, Ha: number of active molecules in the hit list obtained by screening a library with the model under study, FN: false negatives, FP: false positives, %Y : asset return, %A: asset ratio, EF: enrichment factor, GH: hit list goodness.

# Model	D	A	Ht	Ha	FN	FP	%Y	%A	EF	GH
**1**	33	10	1	1	9	0	100	10	3.30	0.77
**2**	33	10	6	6	4	0	100	60	3.30	0.90
**3**	33	10	10	8	2	2	80	80	2.64	0.73
**4**	33	10	23	10	0	13	43.48	100	1.43	0.25
**5**	33	10	16	7	3	9	43.75	70	1.44	0.31
**6**	33	10	17	10	0	7	58.82	100	1.94	0.48
**7**	33	10	16	7	3	9	43.75	70	1.44	0.31
**8**	33	10	21	9	1	12	42.86	90	1.41	0.26
**9**	33	10	23	10	0	13	43.48	100	1.43	0.25
**10**	33	10	23	10	0	13	43.48	100	1.43	0.25
**11**	33	10	23	10	0	13	43.48	100	1.43	0.25
**12**	33	10	16	10	0	6	62.50	100	2.06	0.53
**13**	33	10	15	9	1	6	60	90	1.98	0.50
**14**	33	10	16	10	0	6	62.50	100	2.06	0.53
**15**	33	10	13	8	2	5	61.54	80	2.03	0.52
**16**	33	10	5	3	7	2	60	30	1.98	0.48
**17**	33	10	15	9	1	6	60	90	1.98	0.50
**18**	33	10	14	10	0	4	71.43	100	2.36	0.65
**19**	33	10	14	10	0	4	71.43	100	2.36	0.65
**20**	33	10	13	10	0	3	76.92	100	2.54	0.72
**21**	33	10	12	10	0	2	83.33	100	2.75	0.80
**22**	33	10	12	10	0	2	83.33	100	2.75	0.80
**23**	33	10	11	10	0	1	90.91	100	3.00	0.90
**24**	33	10	10	10	0	0	100	100	3.30	1.00

CONFORMATIONAL CALCULATION OF THE HITS OBTAINED FROM THE PROSPECTIVE VIRTUAL SCREENING IN 2D

The next step in the virtual screening process consisted of calculating the conformation of the 2D prospective virtual screening hits and applying the 3D filter model

number 24. Computation of the 2D hit conformations was performed using the MOE procedure described in the preparation section for 3D filtering.

PROSPECTIVE 3D VIRTUAL SCREENING AND CLUSTERING

For 3D filtering the pharmacophore model number 24 was applied at all times. The statistics for the 3D prospective virtual screening were as follows: Molecular Operating Environment database yielded 30 preliminary hits with 1313 distinct conformations for a total of 660,961 entries, that is a 0.005 % of the library volume. For each 3D hit that conformation with the lowest RMSD value with respect to the pharmacophore model 24 was selected, with the aim of choosing all those conformations with the best-suited spatial requirements given by the pharmacophore model 24. This way we elegantly collected near-active conformations of the hits.

Subsequently, the selected conformations were grouped into clusters based on their similarity (Tanimoto coefficient), with the aim of classifying the results. The clustering protocol was chosen after carrying out several tests, of which the one with the best results was selected. To carry out the above, the TAT-type fingerprints of the 3D hits were calculated, the selection of this type of fingerprint was made since it provides an adequate pharmacophoric coding to facilitate the grouping process, it is calculated from the 3D conformations and to each atom. it is assigned a code according to the following characteristics: donor, acceptor, polar, anion, cation, hydrophobic. The features mentioned above are directly related to the ligand-receptor recognition process. Once the molecules were coded with the TAT fingerprint, they were lumped together using the Tanimoto coefficient, with a similarity percentage of 85% and an overlap of 85%. The literature reports that molecules with a Tanimoto coefficient greater than 85% are considered similar [S47]. Of note, the set percentage was reasonable in a twofold way: (i) in sight of the over half a million data entries, this threshold was devised to reduce the amount considerably; and (ii) the similarity was restricted closer enough to receive answers with an expected higher potency than if the threshold were set with more tolerance. At the end of this stage only 30 molecules remained after 3D screening. They were docked and ranked by the docking score of MOE (data not shown). Due to funding limitations, we finally accepted the offer of only two molecules which were donated and presented here (cf. Acknowledgement section).

ADDITIONAL INFORMATION ABOUT DOCKING

The two final candidates obtained by VS – Fmol and AAmol – have different scaffolds compared to sialic acid and the four neuraminidase inhibitor drugs. As a most valuable asset both bear some of the other pivotal physicochemical patterns to recognize and interact with the binding amino acid residues at the neuraminidase N1 binding site. Thusly, it could be inferred that real potential to improve their inhibitory activities could be expected. After experimental confirmation of both inhibition activities successful patent filing took place [34].

With the final poses from molecular docking theoretical insight can be gained concerning the binding modes between receptor amino acid residues and the ligands under scrutiny. To this end the most populated solution cluster were inspected for the highest affinity poses (lowest free energy of binding). In addition, the computed binding modes with the binding amino acid residues were compared with those of the crystal structure reported in the literature (**Figures 4 to 7**). Proposing simpler chemical structures than oseltamivir and congeners with target affinity was the goal of the preceding step screening a database with small synthetic substances.

3. ADDITIONAL INFORMATION ABOUT ADMET PROFILING

Lipinski´s "rule of five" was implemented as screening parameter "RuleOf5" in the ADMET predictor tool as follows:

- Experimental log P > 5 or MlogP > 4.15 (excessive lipophilicity)
- MWt > 500 (large size)
- HBDH > 5 (too many potential hydrogen bond donors)
- M_NO > 10 (too many potential hydrogen bond acceptors).

Results for AAmol by `ADMET Predictor(TM)`

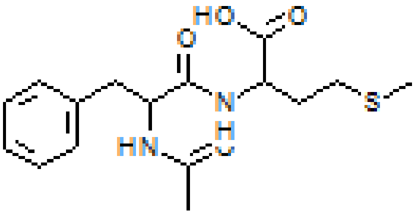

Of note, the double bound oxygen atom label (=O) of the -NHCO-CH_3 is overlapped by the H label and this way only half printed by ADMET Predictor(TM).

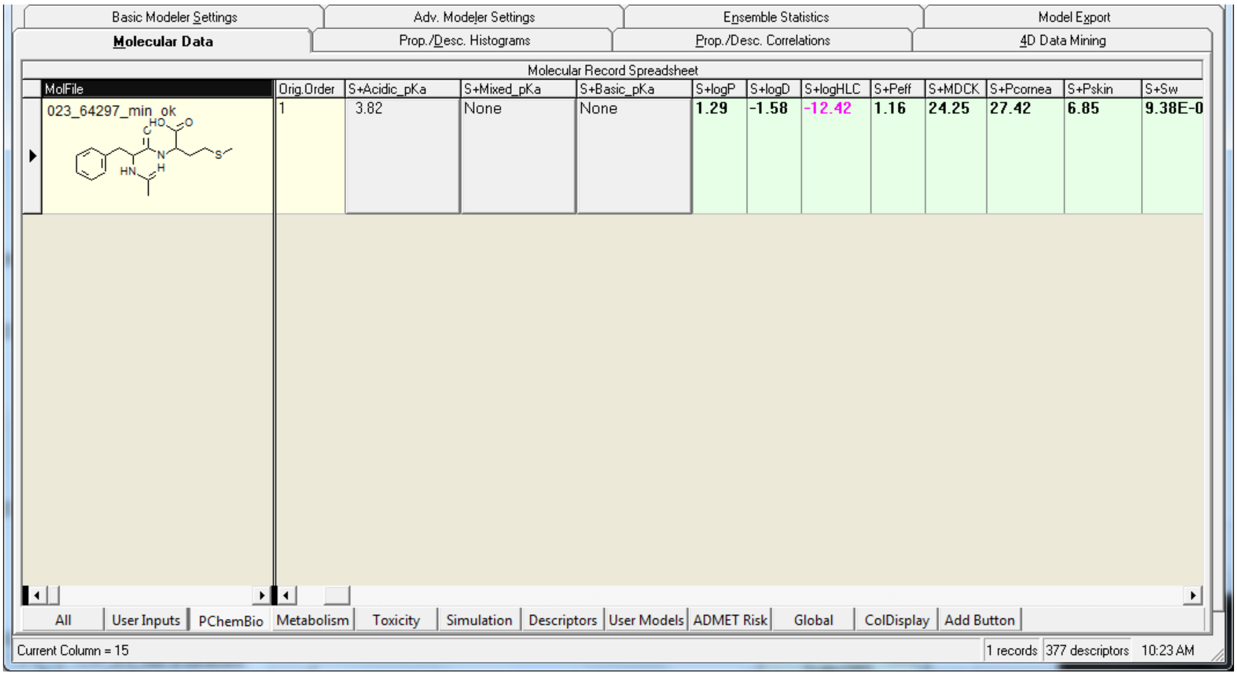

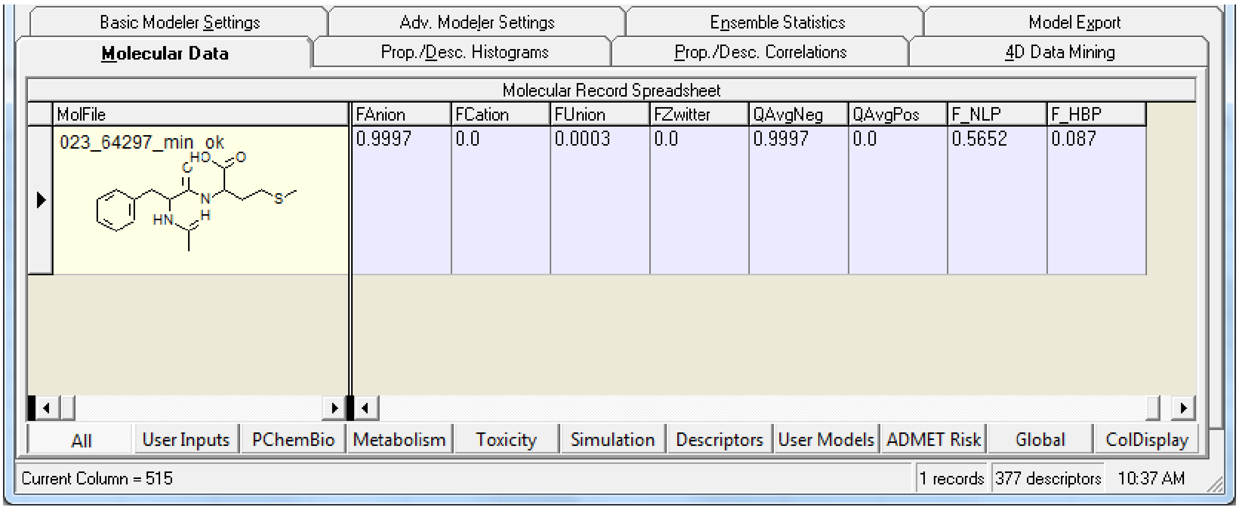

Illustration by two screenshots from the program´s graphical user interface. The GUI is split into two areas for the input (left hand side of the menu) and output (right hand side of the menu). The first screenshot (topmost) shows the leading output features, while the second one (bottommost) shows the final output features for AAmol.

Any numbers in the following schemes present relative ADMET values ordered by imaginary magnitudes for quantitative comparison of a given ADMET feature prediction. A total of 377 ADMET features or descriptors were calculated (Table 3). None of the many features was experimentally validated at this stage of work.

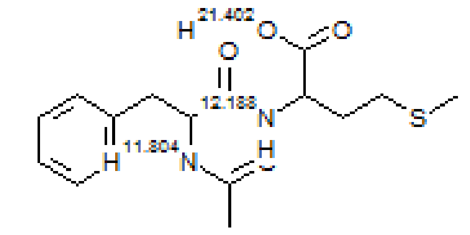

HB acidities

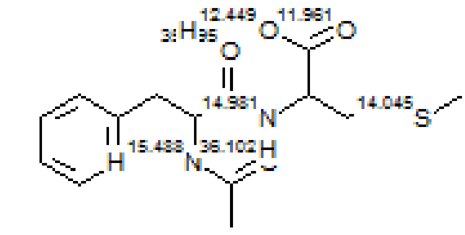

HB basicities

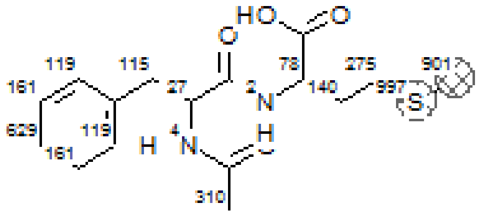

CYP_2C9

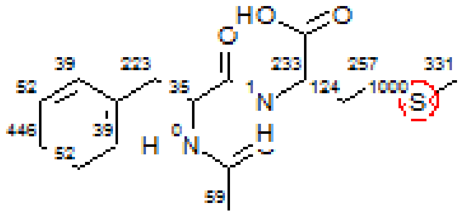

CYP_3A4

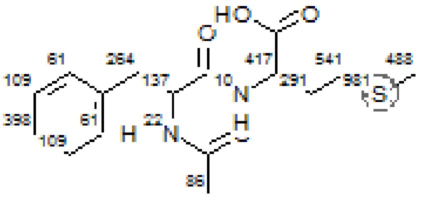

CYP_2A6

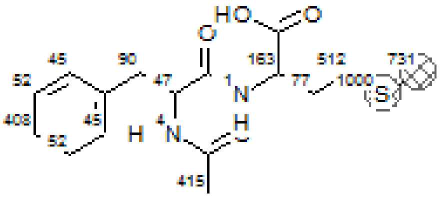

CYP_2B6

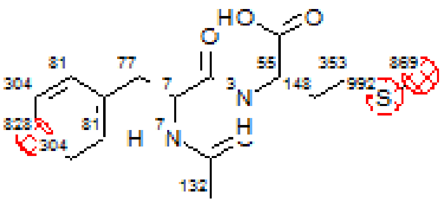

CYP_2C8

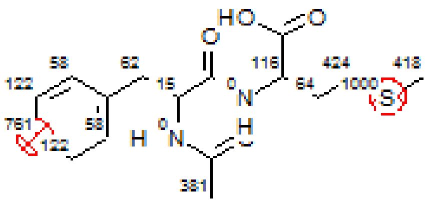

CYP_2C19

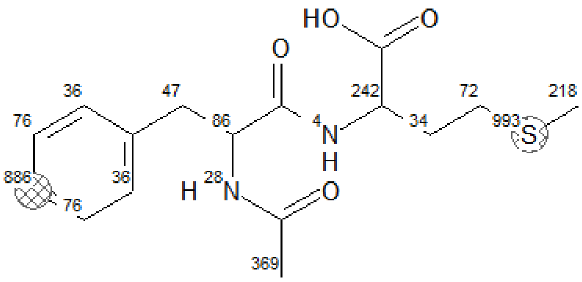

CYP_2D6

Results for Fmol by ADMET Predictor (TM)

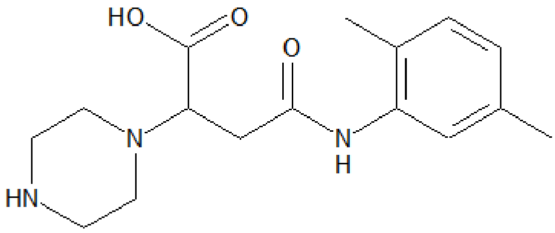

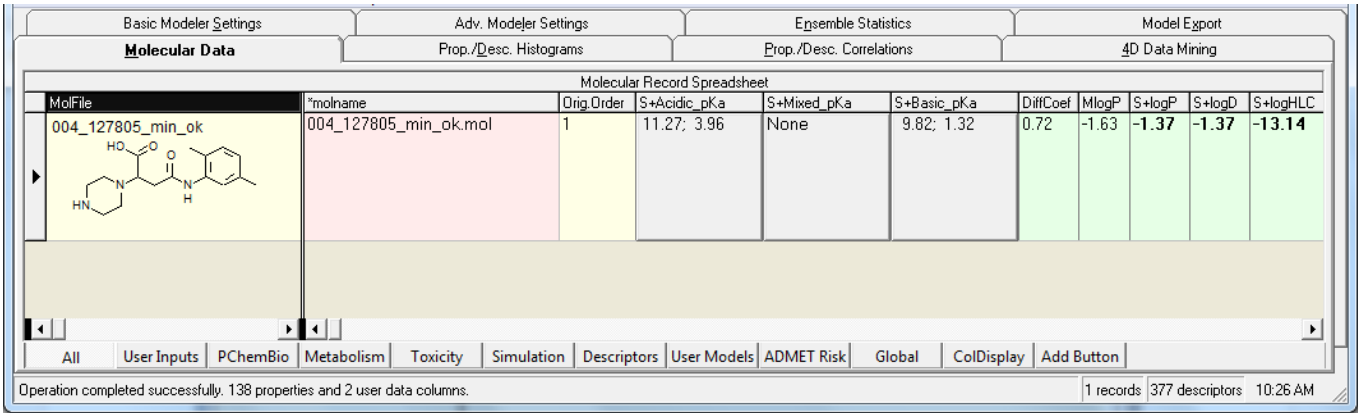

The GUI of ADMET Predictor(TM) screenshot to illustrate the input and output for Fnol.

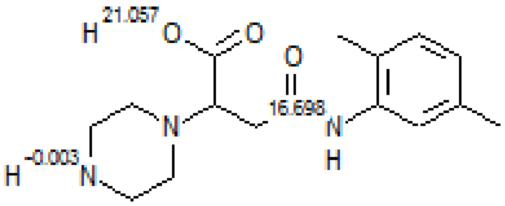

HB acidities

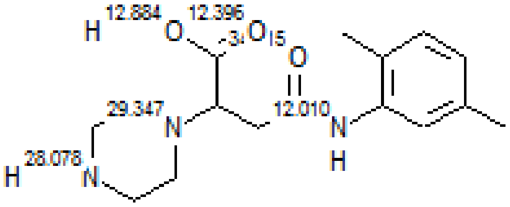

HB basicities

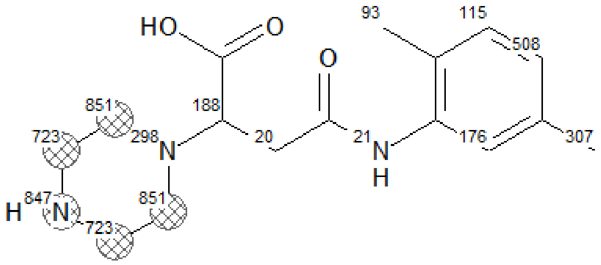

CYP_2A6

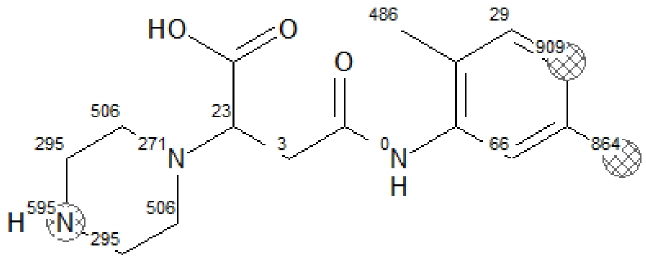

CYP_2C19

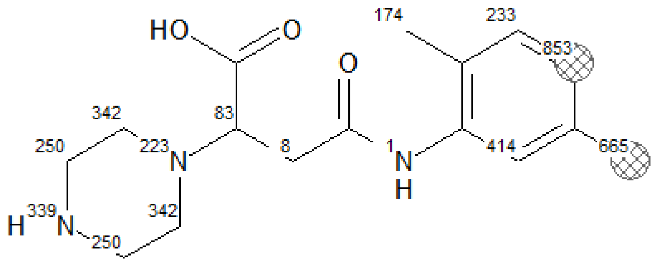

CYP_1A2

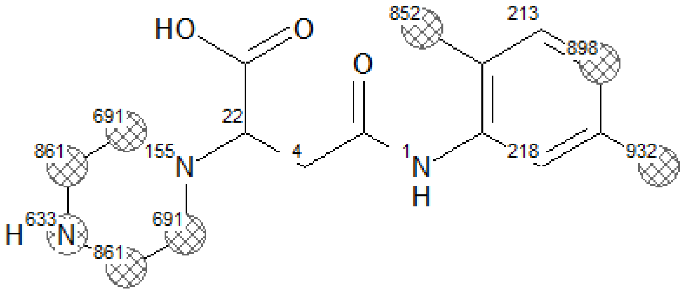

CYP_2C9

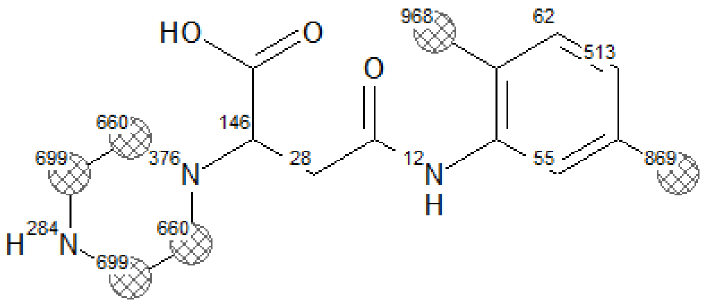

CYP_2E1

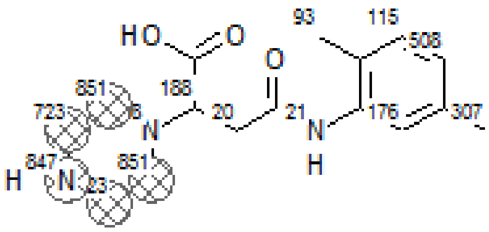

CYP_2A6

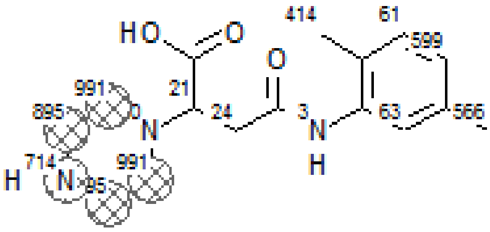

CYP_2B6

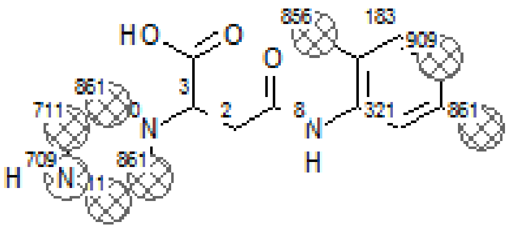

CYP_2C8

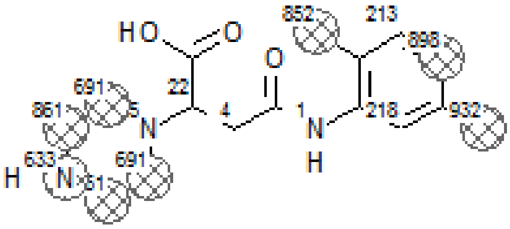

CYP_2C9

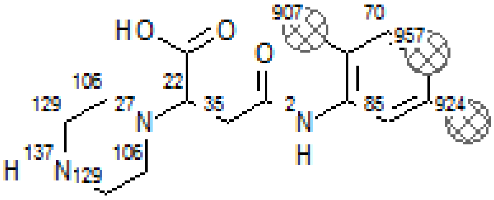

CYP_2D6

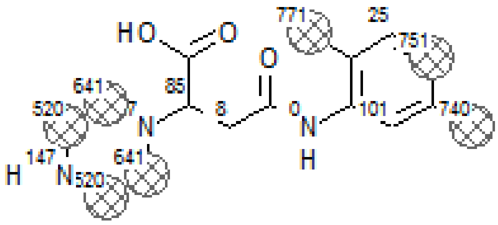

CYP_3A4

REFERNCES FOR SM

[S48]
Kirchmair, J.; Wolber, G.; Laggner, C.; Langer, T. Comparative performance assessment of the conformational model generators omega and catalyst: a large-scale survey on the retrieval of protein-bound ligand conformations. J Chem Inf Model 2006, 46, 1848-1861.

[S49]
Shaillay Kumar, D. Script for computing Tanimoto coefficient. QSARWorld – free online resource for QSAR modeling http://www.qsarworld.com/virtual-workshop.php.
